# Metabolic Profiling of Alpine and Ecuadorian Lichens

**DOI:** 10.3390/molecules201018047

**Published:** 2015-10-01

**Authors:** Verena K. Mittermeier, Nicola Schmitt, Lukas P. M. Volk, Juan Pablo Suárez, Andreas Beck, Wolfgang Eisenreich

**Affiliations:** 1 Lehrstuhl für Biochemie, Technische Universität München, Lichtenberg-Str. 4, D-85747 Garching, Germany; E-Mails: verena.mittermeier@tum.de (V.K.M.); nicola.schmitt1@gmx.de (N.S.); lukas.volk@web.de (L.P.M.V.); 2 Departamento de Ciencias Naturales, Universidad Técnica Particular de Loja, San Cayetano Alto s/n, C.P. 11 01 608 Loja, Ecuador; E-Mail: jpsuarez@utpl.edu.ec; 3 Department of Lichenology and Bryology, Botanische Staatssammlung München, Menzinger Str. 67, D-80638 München, Germany; 4 GeoBio-Center, Ludwig-Maximilians Universität München, Richard-Wagner-Str. 10, D-80333 München, Germany

**Keywords:** metabolomics, principle component analysis, chemotaxonomy, *Sticta*, *Stereocaulon*, sticticin

## Abstract

Non-targeted ^1^H-NMR methods were used to determine metabolite profiles from crude extracts of Alpine and Ecuadorian lichens collected from their natural habitats. In control experiments, the robustness of metabolite detection and quantification was estimated using replicate measurements of *Stereocaulon alpinum* extracts. The deviations in the overall metabolite fingerprints were low when analyzing *S. alpinum* collections from different locations or during different annual and seasonal periods. In contrast, metabolite profiles observed from extracts of different Alpine and Ecuadorian lichens clearly revealed genus- and species-specific profiles. The discriminating functions determining cluster formation in principle component analysis (PCA) were due to differences in the amounts of genus-specific compounds such as sticticin from the *Sticta* species, but also in the amounts of ubiquitous metabolites, such as sugar alcohols or trehalose. However, varying concentrations of these metabolites from the same lichen species e.g., due to different environmental conditions appeared of minor relevance for the overall cluster formation in PCA. The metabolic clusters matched phylogenetic analyses using nuclear ribosomal DNA (nrDNA) internal transcribed spacer (ITS) sequences of lichen mycobionts, as exemplified for the genus *Sticta*. It can be concluded that NMR-based non-targeted metabolic profiling is a useful tool in the chemo-taxonomy of lichens. The same approach could also facilitate the discovery of novel lichen metabolites on a rapid and systematical basis.

## 1. Introduction

Metabolomics is a technology that is based on the non-targeted analysis of small-sized metabolic intermediates or products in complex biological fluids or crude extracts using state-of-the-art spectroscopy on a quantitative basis (e.g., GC/MS, LC/MS and ^1^H-NMR). The various methods can provide complementary data and, therefore, a combination of MS- and NMR-based fingerprints results in the most comprehensive set of metabolites. Typically, the spectroscopic fingerprints are then interpreted by multivariate statistical methods, such as principle component analysis (PCA) in conjunction with pattern recognition and signal assignment techniques. As a result of this analysis, specific metabolic sets or single metabolic markers are correlated with the genetic backgrounds and/or environmental factors. For example, metabolomics has been applied to provide the basis for chemo-systematic analyses of microorganisms, to discover new natural products, to elucidate metabolic fluxes in the organisms under study, to better understand the dynamics of metabolic networks in response to abiotic or biotic triggers, or to identify novel markers in the diagnosis of disease (for recent reviews, see [1,2,3]).

It can be assumed that these metabolomics techniques also benefit the analysis of lichens on the basis of their unique metabolic compositions. Indeed, hundreds of lichen-specific metabolites have been isolated and characterized during the last decades by classical chromatographic and spectroscopic methods [4,5,6]. Most of these lichen compounds belong to polyketides, polyphenols, quinones and terpenoids, presumably of fungal origin. On the other hand, well-known and ubiquitous sugars and sugar alcohols (e.g., mannitol, arabitol, ribitol, and sucrose) have been described as abundant components in many lichen species under study (for a review, see [7]). Interestingly, sugar alcohols were found at increased concentrations when the same lichens were collected under dry conditions [8]. Therefore, the chemo-taxonomy of field-collected lichens on the basis of non-targeted methods has to consider environmental factors that can hardly be controlled. Thus, it is no surprise that multivariate non-targeted methods have only rarely been used to characterize lichens on the basis of specific metabolite profiles [9].

In this paper, we have now used NMR-based metabolomics to assess the power and limits of the non-targeted methodology in the analysis of lichens. In a pilot experiment, we have first analyzed the reproducibility of metabolic fingerprints from the Alpine lichen, *Stereocaulon alpinum* Laurer, collected at different habitats, heights, seasons, and external conditions. Optimized protocols were then developed and used to characterize a set of Ecuadorian lichens with a special focus on *Sticta* species. Multivariate analysis revealed the specificity of the metabolic fingerprints of most lichens under study. Some of the genus-specific metabolic markers could subsequently be identified by multi-dimensional NMR spectroscopy of the crude extracts without prior purification. The relevance of the study was underlined by comparison of cluster formation due to metabolic and genomic patterns, as exemplified for an array of *Sticta* species.

## 2. Results and Discussion

### 2.1. Metabolic Fingerprinting of Lichens

#### 2.1.1. Optimization of Extraction Protocols and Analytical Methods for the Non-Targeted Analysis of Lichens

Selecting and controlling the parameters in collecting, storing, and extracting the organisms under study is crucial when performing non-targeted, unbiased metabolomic analyses, since these steps are known to have fundamental effects on the metabolite profiles, as shown by many studies with microorganisms or higher organisms. Using a given collection of the Alpine lichen, *Stereocaulon alpinum*, we, therefore, first compared metabolite profiles in extracts using various solvents and procedures including extraction under reflux or at room temperature, or applying mechanical cell disruption.

For this purpose, we extracted standard amounts (30 mg, dry weight) of *S. alpinum* under reflux or in the cold using methanol or hexane as solvent. Alternatively, we mechanically disrupted the same amount suspended in methanol. The extracts were then subjected to ^1^H-NMR spectroscopy at 27 °C using repetition rates of about 10 s and 30° pulses in the NMR experiment. The NMR spectra of each of these samples showed well-defined patterns including sharp signals with applicable signal-to-noise ratios. Thus, the same amount of lichen thallus mass (30 mg, dry weight) was used in the following analyses. Replicate NMR measurements of extracts from the same sample displayed virtually identical spectra indicating the robustness of the quantitative NMR approach and the validity of the parameters in the measurements. Moreover, the ^1^H-NMR spectra of *S. alpinum* samples obtained by the different extraction procedures were also highly similar when using a given solvent (*i.e.*, methanol or hexane) indicating that the principle compounds characterizing the NMR profiles were obtained at similar amounts by each of the used methods. On this basis, we extracted all lichen samples described below with hexane or methanol, respectively, under reflux for 40 min (hexane) or 20 min (methanol). We are aware that this procedure gives rise to complete or partial degradation of some metabolic intermediates, such as sugar phosphates. However, since we focused our analysis on stable metabolites, this protocol appeared applicable.

#### 2.1.2. Quality Control: Reproducibility of Metabolic Profiles in Extracts of *Stereocaulon alpinum*

In the next step, we compared the ^1^H-NMR fingerprints of these extracts using collections of *S. alpinum* from different locations, years or seasons. Whereas the spectra of the hexane extracts were virtually identical displaying major signals due to the presence of atranorin [10], the spectra of the methanolic extracts were qualitatively similar, but some signals in the spectral region between three and five ppm tentatively assigned to sugars or sugar alcohols were different in their relative sizes. Notably, the signal patterns in the spectral region between five and 10 ppm were identical in all of the samples under study. It can be concluded that multivariate analyses based on NMR signal patterns between three and five ppm in the methanolic extracts could reveal environmental factors such as dryness, whereas analyses using patterns in the hexane extracts or the down-field NMR-region of the methanolic extracts could reflect genus-specific profiles.

### 2.2. Comparative Metabolic Profiling of Different Ecuadorian Lichens

#### Metabolic Fingerprinting by ^1^H-NMR

##### Hexane Extracts

**Figure 1:**
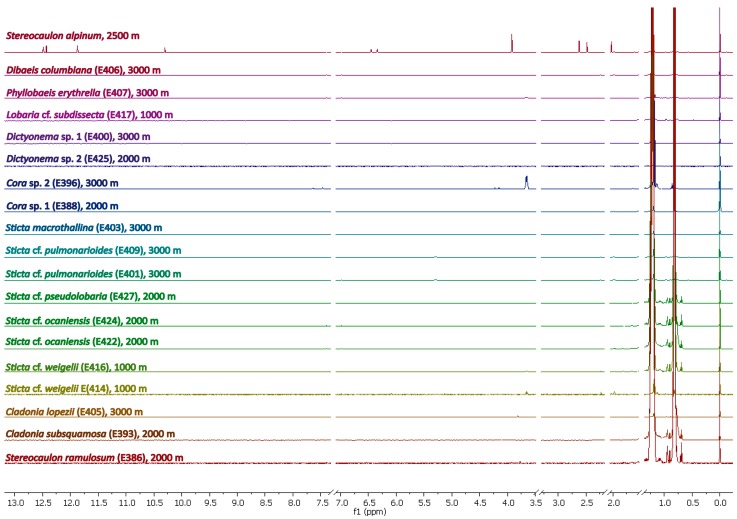
^1^H-NMR spectra of hexane extracts of *S. alpinum* (top trace) and some Ecuadorian lichens between 0.0 and 13.0 ppm. “Unspecific” signals due to solvents were omitted. Spectra were scaled to the signal intensity of CHCl_3_ from the solvent.

Figure 1 shows the ^1^H-NMR spectra of the hexane extracts of some Ecuadorian lichens collected at different heights above sea-level. The spectra were sorted by the genera of lichens and the heights at which they were collected. The signals around 0.8 ppm and 1.2 ppm were due to impurities from the solvent and were therefore not considered in the subsequent comparative analyses. Since most of the hexane extracts exhibited only weak NMR signals, the Ecuadorian lichens under investigation contained hydrophobic substances only at low concentrations regardless of their species or habitat. Notably, this was in sharp contrast to the hexane extracts from *S. alpinum* displaying intense signals due to atranorin (top trace in Figure 1). As an exception of Ecuadorian lichens, some minor down-field shifted signals were detected in the extract of *Cora* sp. 2 collected at 3000 m, but not in *Cora* sp. 1 from the habitat at 2000 m.

##### Methanolic Extracts

**Figure 2:**
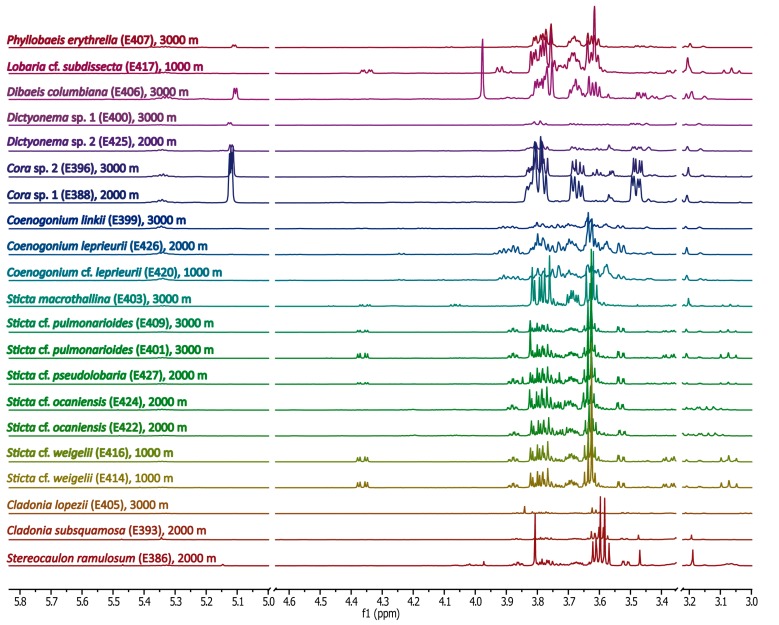
^1^H-NMR spectra of methanolic lichen extracts between 3.0 and 5.8 ppm. The residual signals of water (δ = 4.8 ppm) and the solvent (δ = 3.3 ppm) are not shown. Spectra were scaled to the intensity of the residual methyl signal of the MeOD solvent.

**Figure 3:**
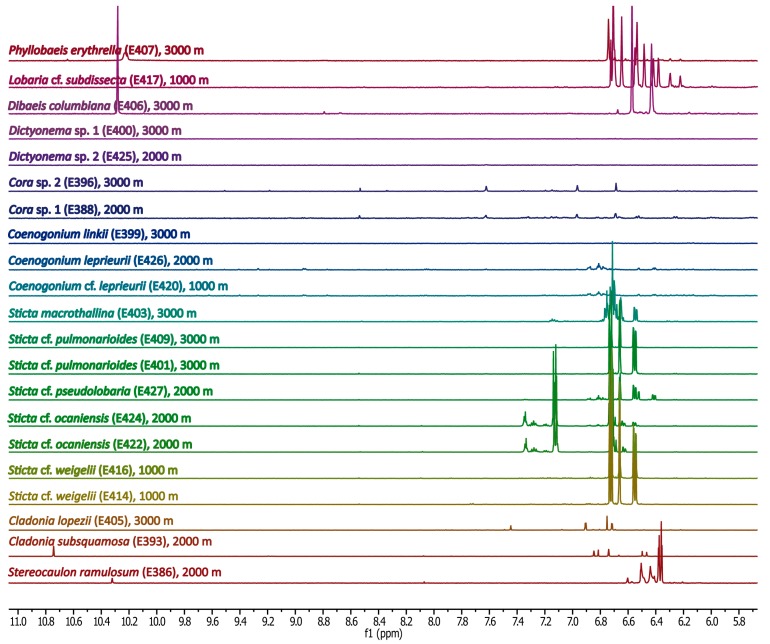
^1^H-NMR spectra of methanolic lichen extracts between 5.8 and 11.0 ppm. In comparison to the signal heights in Figure 2, the signals were increased by a factor of seven.

Figure 2 shows the ^1^H-NMR spectra of the methanolic extracts of Ecuadorian lichen samples between 5.8 and 3.0 ppm. The spectra were again sorted as in Figure 1. Many signals with high intensities were observed in this spectral region indicating the presence of sugars or sugar alcohols. For all lichen genera and species, the signals in the spectral region between 11.0 and 5.8 ppm (Figure 3) were much lower in intensity compared with the up-field NMR region. Notably, a clear distinction can be seen between all genera, and even species under study, on the basis of the ^1^H-NMR patterns in both spectral regions.

### 2.3. Multivariate Analysis: Comparison of Ecuadorian and Some Alpine Lichens by Principle Component Analysis

**Figure 4:**
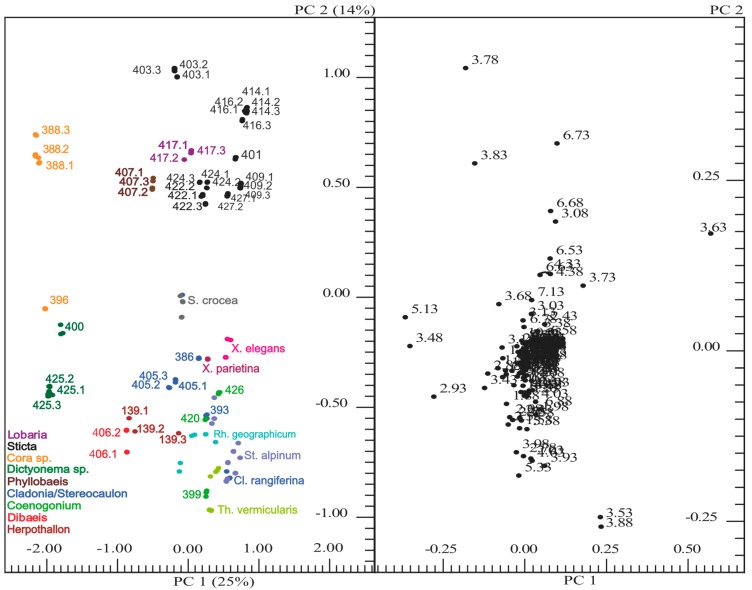
Scores plot (on the left side) and loadings plot (on the right side) of Ecuadorian and some Alpine lichens. Scores plot: each spot represents one ^1^H-NMR spectrum (three technical replicates of each sample). Biological replicates are distinguished by the following way: 388.1 designates the first biological replicate of lichen #388. Loadings plot: each dot denotes the mean value of the bucket.

The ^1^H-NMR spectra of the methanolic extracts from the Ecuadorian lichens and some Alpine lichens were digitized into small sections (“buckets”) and statistically analyzed using principal component analysis (PCA). The changes in the metabolite composition are visualized in the two-dimensional scores plot (Figure 4, left). The plot is composed of PC1 (x-axis) and PC2 (y-axis), partly explaining the variance of the spectra. Each point in the plot represents a given NMR spectrum and therefore an individual lichen sample. If enough material was available, three individual extracts were obtained from a given lichen collection and the resulting spectra were subjected to the PCA analysis. The close neighborhood of these replicates in the scores plot (Figure 4, left) confirmed the validity of the method. Furthermore, closely related species such as *Xanthoria elegans* and *Xanthoria parietina* or *Lobaria* and *Sticta* lichens, belonging to the same family, respectively, were also closely neighbored in the scores plot. On the other hand, the distribution of the spectra in the scores plot showed that *Sticta*, *Lobaria*, *Phyllobaeis*, and *Cora* were clearly separated from the other lichens in PC2. Moreover, PC2 partly separated the two basidiolichen genera *Cora* and *Dictyonema*.

Visual inspection and comparison of the NMR spectra had already indicated that the metabolite composition was at least not greatly affected by the growth location (see above). This conclusion was confirmed by the fact that no clear separation between Alpine and Ecuadorian lichens could be observed. Rather, the detected clusters in the scores plot indicated that the profiles were mostly determined by the genus and/or family of the lichen under study.

To investigate the specific differences in the scores plot, the loadings plot (Figure 4, right) was now examined. The separation of both *Cora*/*Dictyonema* species was caused by buckets at 5.13, 3.88, 3.83, 3.78, 3.63, 3.53, 3.48, and 2.93 ppm, which were tentatively assigned to ^1^H-NMR signals of trehalose, arabitol, mannitol, ribitol, and ornithine [11]. Indeed, both species contained lower amounts of sugar alcohols, but higher amounts of trehalose in comparison to the other lichens under study (see below). The peaks at 6.73, 6.68, 6.53, and 3.08 ppm explained the separation between *Sticta*, *Lobaria*, *Phyllobaeis*, and the other lichens. These buckets covered the signals for sticticin [12] in *Sticta* and *Lobaria*, and probably norstictic acid, in *Phyllobaeis* [13].

### 2.4. Identification of Marker Compounds by NMR

#### 2.4.1. Sugars and Sugar Alcohols

**Figure 5:**
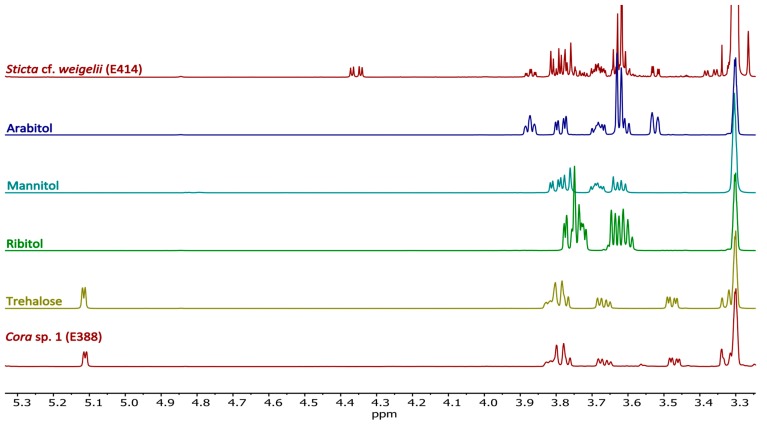
^1^H-NMR spectra of different reference compounds in comparison with methanolic lichen extracts of *Sticta* and *Cora*.

In order to verify some of the tentative ^1^H-NMR assignments for sugars and sugar alcohols, we compared the complex NMR data with authentic reference compounds, e.g., trehalose, mannitol, ribitol, and arabitol measured under the same conditions as for the crude extracts, *i.e.*, using deuterated methanol as solvent. This was especially important since the available spectral NMR libraries are based on measurements in D_2_O. Due to the specific signal patterns of these compounds, the comparisons resulted in solid assignments (Figure 5), as exemplified for trehalose in #E388 (*Cora* sp. 1) and arabitol and mannitol in #E414 (*Sticta cf. weigelii* Isert), also confirming the GC-MS analyses described below.

#### 2.4.2. Lichen-Specific Compounds

**Figure 6:**
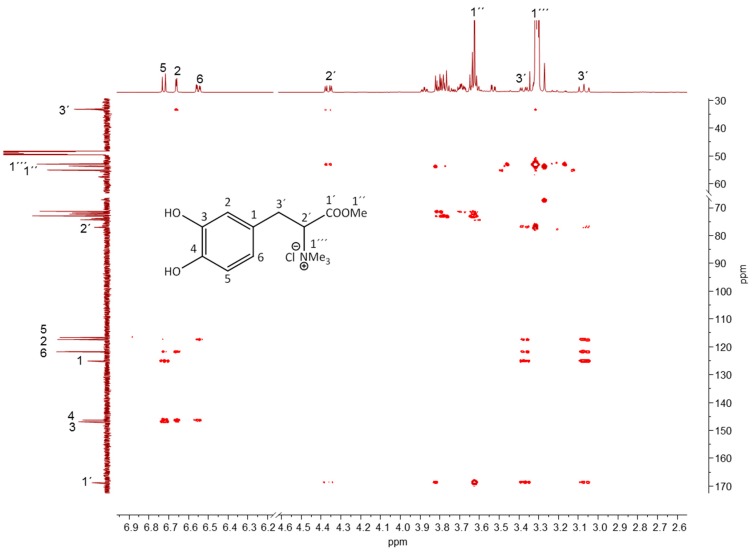
Two-dimensional NMR analysis of crude lichen extracts. Section of the HMBC spectrum of the methanolic extract from #E414 (*Sticta cf. weigelii*). The structure of sticticin is shown in the inset. ^13^C- and ^1^H-NMR spectra of the crude extract of #E414 are shown as projections with assignments of the sticticin signals.

Many of the major signals detected in the crude extracts of the lichens under investigation could not be easily assigned on the basis of the observed chemical shifts and patterns. This was expected since many of the specific lichen compounds are not found in the standard NMR libraries. Especially metabolites causing signals due to aromatic compounds could not be assigned to a known structure. Therefore, we have performed two-dimensional NMR experiments using the crude extracts without prior purification to enable structure determination of the major compounds, *i.e.*, causing the main signals that could not be assigned by comparison with reference materials. As an example, Figure 6 shows the two-dimensional HMBC spectrum of the methanolic extract of #E414 (*Sticta cf. weigelii*). The chemical shifts and the coupling patterns of the signals in the “aromatic region” clearly indicated that one of the major metabolites in the *Sticta* extract was a 1,3,4-substituted aromatic compound. The detected correlation patterns in conjunction with COSY, NOESY, and HMBC data (Table 1) left no doubt that the major signals marked in Figure 6 were due to the presence of sticticin, a well-known alkaloid in *Sticta* species [5,12].

**Figure 7:**
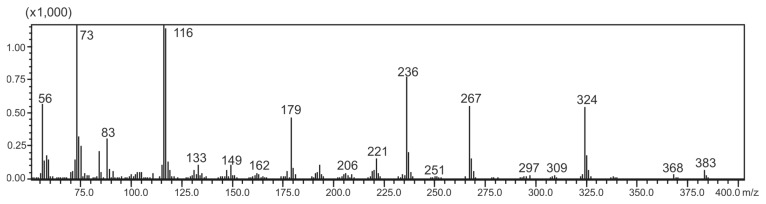
Mass spectrum of disilylated sticticin.

**Figure 8:**
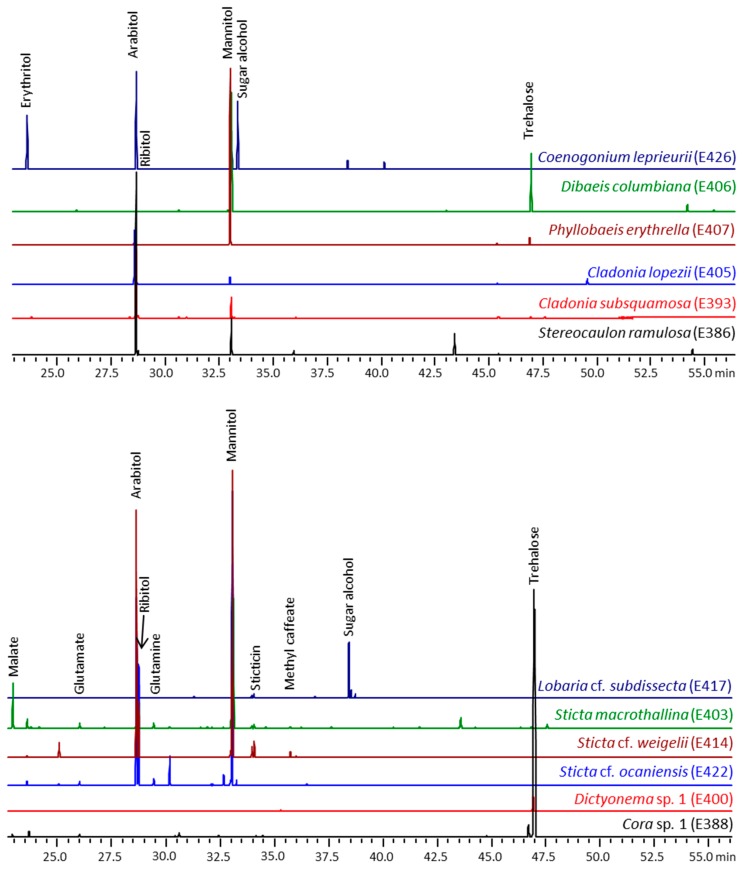
Detection of metabolites (as MSTFA derivatives) by GC-MS in methanolic extracts of some Ecuadorian lichens. The compounds were assigned on the basis of their specific MS patterns by comparison with reference data. The chromatograms were scaled to the peak height of the norvaline standard.

The assignment was further confirmed by a close inspection of the detected peak at 33.97 min in the GC-MS run of the sample (Figure 7 and Figure 8; Table 2), as well as by the conversion of sticticin into caffeic acid methyl ester after adding sodium hydroxide to the crude extract of #E414. In the hydrolysate, the characteristic methylene groups of methyl caffeate were detected (data not shown).

**Table 1:** NMR data of sticticin on the basis of the detected signals using the crude extract of #E414 (*Sticta cf. weigelii*).

Position	^1^H (ppm)	Integral	Multiplicity	*J*_H,H_^a^ (Hz)	^13^C (ppm)	Correlations Observed		
						COSY	HMBC	NOESY
5	6.72	1	d	8.1 (6)	116.67	H(6)		
2	6.66	1	d	2.1 (6)	117.42		H(3′), H(5), H(6)	H(3′)
6	6.55	1	dd	8.1 (5); 2.1 (2)	121.79	H(5)	H(3′), H(5), H(2)	H(3′)
2′	4.36	1	dd	12.1 (3′); 4.1 (3′)	77.06	H(3′)	H(3′)	
1″	3.63	1	s		53.56			
1′′′	3.32	nd	s		52.97		H(2′)	
3′	3.38	1	dd	12.5 (3′); 4.1 (2′)	33.35	H(2′), H(3′)	H(2), H(2′), H(1′′′)	
3′	3.07	1	t	12.1 (2′)	33.35	H(2′), H(3′)	H(2), H(2′), H(1′′′)	
1′					164.74		H(3′), H(2′), H(1″)	
3					146.88		H(5), H(2)	
4					146.29		H(5), H(2), H(6)	
1					125.05		H(5), H(3′)	

^a^ Coupling partners are given in parentheses; nd, not determined due to signal overlap; d, doublet; dd, doublet of doublets; s, singlet and t, triplet.

**Table 2:** MS data of disilylated sticticin.

Fragment Ion	*m*/*z*
Disilylated sticticin-CH_3_Cl [M]^+^	383
[M − CH_3_]^+^	368
[M − C(1′)OOCH_3_]^+^	324
[M − C(1′)OOCH_3_C(2′)HN(CH_3_)_2_]^+^	267
[C(1′)OOCH_3_C(2′)HN(CH_3_)_2_]^+^	116
[Si(CH_3_)_3_]^+^	73

### 2.5. Identification of Marker Compounds by GC-MS

To further identify key metabolites in the methanolic extracts, the metabolite profiling was now accomplished by GC/MS using the MSTFA-derivatives of the NMR samples. Figure 8 shows chromatograms of representative runs illustrating that all lichens except of *Cora*/*Dictyonema* species contained mannitol and arabitol at high concentrations. Furthermore, the photobiont-specific polyols, ribitol, and erythritol were detected in some of the metabolite profiles. Ribitol was reported as the main carbon transfer substance in lichens with *Asterochloris* (*Cladonia*, *Stereocaulon*), *Elliptochloris* (*Phyllobaeis*), and *Coccomyxa* (*Dibaeis*), and erythritol in lichens with *Trentepohlia* (*Coenogonium*) [14]. The high concentration of trehalose in *Cora*/*Dictyonema* could reflect that glucose is exported in lichens with cyanobacteria, since trehalase converts this non-reducing disaccharide into two molecules of glucose.

In addition, the comparison of *Sticta* samples showed that mannitol was the most abundant metabolite in wet lichens, whereas higher amounts of arabitol than mannitol were found in lichens collected under dry conditions (for example, #E422 as shown in Figure 8, but also #E424). Indeed, these differences were also evident in the PCA scores plot (Figure 4). These results confirmed that mannitol may protect lichens against water loss, whereas arabitol is a more readily mobilizable reserve [15].

In contrast to sugars and sugar alcohols, free amino acids were only extracted at low concentrations. Among amino acids, glutamine, glutamate, and alanine were the most abundant ones. The relatively high level of glutamate and glutamine supports the suggestion that they function as main nitrogen storage compounds in lichens [16].

### 2.6. Taxon Analyses with Molecular Methods

**Figure 9:**
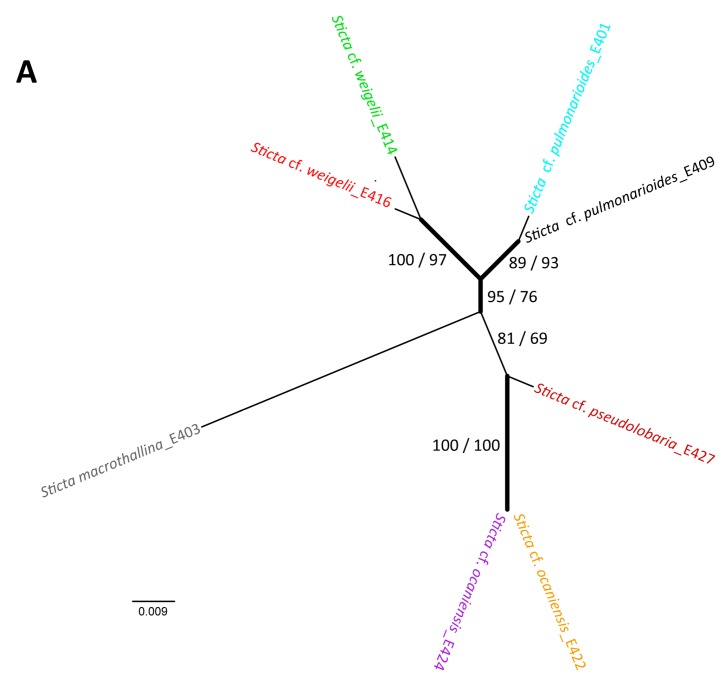

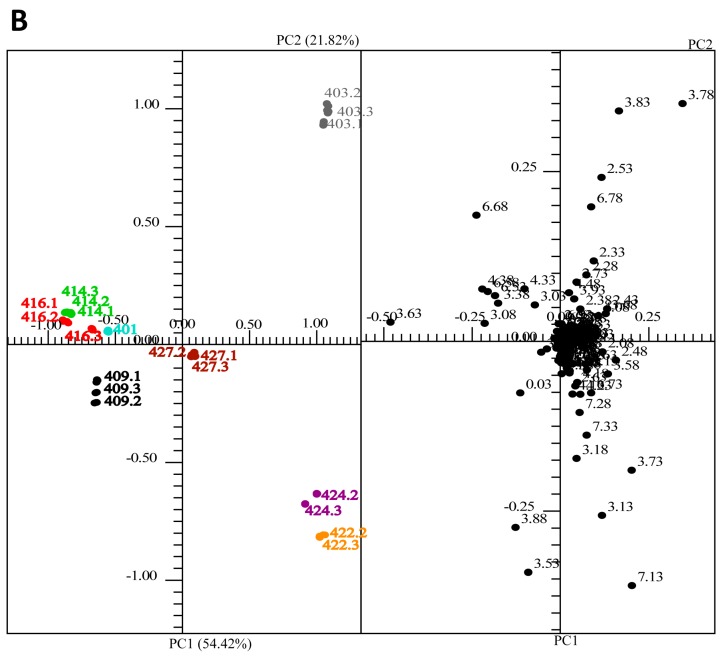
nrITS DNA phylogeny (**A**) and PCA of the lichen metabolites in the methanolic extracts (**B**) for representative specimens of the genus *Sticta*. Corresponding colors were used to indicate the same lichen species in (**A**) and (**B**).

In order to evaluate the genetic diversity of the sampled *Sticta* specimens, molecular investigations of the mycobiont ITS nrDNA region have been conducted as described in Section 3.3. The resulting phylogram (Figure 9A) depicts similar groupings like the multivariate analysis of the metabolic content in the methanolic extracts of these specimens (Figure 9B). The genetically and morphologically most peculiar species is *S. macrothallina* Moncada and Coca (#E403), recognized by its much broader lobes. The specimens of *S. cf. ocaniensis* Moncada and Simijaca (#E422, #E424) group together and form a branch including the closely related *S. cf. pseudolobaria* Moncada and Coca (#E427)—both belonging to the *S. canariensis*-clade [17]—but with low support only. All three specimens are located within the same subsquare in the PCA analysis as well. The specimens of *S. pulmonarioides* Moncada and Coca (#E401, #E409) form a well-supported group with the specimens of *S. cf. weigelii* (#E414, #E416). Again these groups are also represented in the PCA analysis of metabolic compounds, with the minor exception of *S. pulmonarioides* (#E401) which seems closer to *S. cf. weigelii* regarding its metabolic compounds, but due to material restrictions only one biological replicate could be analyzed and all samples from these lichens are grouped in the left half of the PCA graph.

## 3. Experimental Section

### 3.1. Chemicals

The following compounds were obtained commercially from the suppliers given in brackets:

l-arabitol, *meso*-erythritol (Alfa Aesar, Karlsruhe, Germany); *n*-hexane (98.2% purity; Merck, Darmstadt, Germany); methanol (99.8% purity; VWR, Leuven, Belgium); d-mannitol, l-ornithine, ribitol, d-trehalose (Sigma-Aldrich, Steinheim, Germany). Deuterated solvents were obtained from Euriso-Top (Gif-Sur-Yvette, France).

### 3.2. Lichen Collection and Storage

Lichens have been collected in Southern Ecuador in August 2012 or January 2011 (AB_E139). Environmental factors for the different altitudinal collecting sites in Ecuador are given in detail by Bendix *et al.* [18]. Alpine lichens were collected in Tyrol (Obergurgl, Austria) in 15th of September 2012 (cloudy) from heights of 2.000–2.700 m a.s.l. (temperature, 10–14 °C). Collecting details are given in Table 3.

**Table 3:** Collection details for the lichen specimens of this study.

Species	Col. Nr.	Locality	Col. Date
*Cladonia lopezii*	AB_E405	Podocarpus National Park, Cajanuma; *ca.* 3000 m a.s.l.	17 August.2012
*Cladonia rangiferina*		Zirbenwald, Obergurgl, *ca.* 2.100 m a.s.l.	15 September 2012
*Cladonia subsquamosa*	AB_E393	Podocarpus National Park, San Francisco; *ca.* 2000 m a.s.l.	16 August 2012
*Coenogonium cf. leprieurii*	AB_E420	Podocarpus National Park, Bombuscaro; *ca.* 1000 m a.s.l.	20 August 2012
*Coenogonium leprieurii*	AB_E426	Podocarpus National Park, San Francisco; *ca.* 2000 m a.s.l.	21 August 2012
*Coenogonium linkii*	AB_E399	Podocarpus National Park, Cajanuma; *ca.* 3000 m a.s.l.	17 August 2012
*Dibaeis columbiana*	AB_E406	Podocarpus National Park, Cajanuma; *ca.* 3000 m a.s.l.	17 August 2012
*Cora* sp. 1	AB_E388	Podocarpus National Park, San Francisco; *ca.* 2000 m a.s.l.	16 August 2012
*Cora* sp. 2	AB_E396	Podocarpus National Park, Cajanuma; *ca.* 3000 m a.s.l.	17 August 2012
*Dictyonema* sp. 1	AB_E400	Podocarpus National Park, Cajanuma; *ca.* 3000 m a.s.l.	17 August 2012
*Dictyonema* sp. 2	AB_E425	Podocarpus National Park, San Francisco; *ca.* 2000 m a.s.l.	21 August 2012
*Herpothallon* sp.	AB_E139	Podocarpus National Park, San Francisco; *ca.* 2000 m a.s.l.	22 January 2011
*Lobaria cf. subdissecta*	AB_E417	Podocarpus National Park, Bombuscaro; *ca.* 1000 m a.s.l.	20 August 2012
*Phyllobaeis erythrella*	AB_E407	Podocarpus National Park, Cajanuma; *ca.* 3000 m a.s.l.	17 August 2012
*Rhizocarpon geographicum*		Rotmoostal, Obergurgl, *ca.* 2300 m a.s.l.	15 September 2012
*Solorina crocea*		Zirbenwald, Obergurgl, *ca.* 2100 m a.s.l.	15 September 2012
*Stereocaulon alpinum*		Rotmoosferner, Obergurgl, *ca.* 2700 m a.s.l.	15 September 2012
*Stereocaulon ramulosum*	AB_E386	Podocarpus National Park, San Francisco; *ca.* 2000 m a.s.l.	16 August 2012
*Sticta macrothallina*	AB_E403	Podocarpus National Park, Cajanuma; *ca.* 3000 m a.s.l.	17 August 2012
*Sticta cf. ocaniensis*	AB_E422	Podocarpus National Park, San Francisco; *ca.* 2000 m a.s.l.	21 August 2012
*Sticta cf. ocaniensis*	AB_E424	Podocarpus National Park, San Francisco; *ca.* 2000 m a.s.l.	21 August 2012
*Sticta cf. pseudolobaria*	AB_E427	Podocarpus National Park, San Francisco; *ca.* 2000 m a.s.l.	21 August 2012
*Sticta cf. pulmonarioides*	AB_E401	Podocarpus National Park, Cajanuma; *ca.* 3000 m a.s.l.	17 August 2012
*Sticta cf. pulmonarioides*	AB_E409	Podocarpus National Park, Cajanuma; *ca.* 3000 m a.s.l.	17 August 2012
*Sticta cf. weigelii*	AB_E414	Podocarpus National Park, Bombuscaro; *ca.* 1000 m a.s.l.	19 August 2012
*Sticta cf. weigelii*	AB_E416	Podocarpus National Park, Bombuscaro; *ca.* 1000 m a.s.l.	20 August 2012
*Thamnolia vermicularis*		Rotmoosferner, Obergurgl, *ca.* 2700 m a.s.l.	15 September 2012
*Xanthoria elegans*		Rotmoostal, Orbergurgl, *ca.* 1700 m a.s.l.	15 September 2012
*Xanthoria parietina*		Rotmoostal, Orbergurgl, *ca.* 1500 m a.s.l.	15 September 2012

### 3.3. Taxon Analyses

In order to evaluate the genetic diversity of the sampled *Sticta* specimens molecular investigations of the mycobiont ITS nrDNA region have been conducted as described in Beck and Mayr [19]. Basically, DNA has been isolated using the PCR Template Preparation Kit from Roche (Cat No. 11 796 828 001) following the manufacturers protocol, but grinding the lichen carefully using a micro–pestle in liquid nitrogen before adding the lysis buffer. 10–50 mg of air-dried lichen material has been used for the DNA extraction. 1 µL of a 1:20 dilution of the obtained DNA solution has been used for PCR using fungal primers and ITS4m. All PCR products were purified with the Macherey–Nagel columns (Macherey–Nagel, Düren, Germany) and labeled with Big Dye Terminator v3.1 Kit (Applied Biosystems). Cycle sequencing was 30 cycles of: 95 °C for 10 s, 50 °C for 15 s, 60 °C for 3 min. Post-sequencing cleanup was performed using gelfiltration with Sephadex G–50 Superfine (GE Healthcare, Freiburg, Germany; Cat. No. 17–0041–01) following the manufacturer’s protocol. Forward and reverse strand sequences were detected in an ABI 3730 48 capillary automatic sequencer (Applied Biosystems) and assembled using the Staden package [20]. Double-stranded sequences were aligned manually using Gendoc [21]. All sequences used are detailed in Table 4. Maximum Parsimony (MP) and Maximum Likelihood (ML) searches were conducted using the program PAUP Version 4.0b10 [22]. All characters of the ITS1 and ITS2 region, but not the 5.8S nrDNA, have been included for these calculations. To select the nucleotide substitution model and parameters for the ML searches, a hierarchical likelihood ratio test was carried out as implemented in jModelTest 2.1.5 [23,24]. The optimal model was selected under the Akaike information criterion (TrN+G). Heuristic searches have been conducted with 10,000 random–addition–sequence (RAS) replicates, TBR (tree bisection reconnection) branch swapping, Multrees option in effect, saving all trees and collapsing branches with maximum length equal to zero. Statistical support in all trees was assessed by bootstrap analysis (BS; [25]) using 10,000 bootstrap replicates with five random–addition sequences per replicate, but with the multree option not in effect.

**Table 4:** Samples used for the molecular phylogeny in this study including GenBank accession numbers.

Species	Col. Nr.	Genbank Nr.
*Sticta macrothallina*	AB_E403	KT750878
*Sticta cf. ocaniensis*	AB_E422	KT750880
*Sticta cf. ocaniensis*	AB_E424	KT750881
*Sticta cf. pseudolobaria*	AB_E427	KT750879
*Sticta cf. pulmonarioides*	AB_E401	KT750876
*Sticta cf. pulmonarioides*	AB_E409	KT750877
*Sticta cf. weigelii*	AB_E414	KT750874
*Sticta cf. weigelii*	AB_E416	KT750875

### 3.4. Extraction Methods

#### 3.4.1. Hexane Extraction

Ground lichens were extracted in 5 mL *n-*hexane for 40 min at 65 °C or at room temperature for 24 h. After filtration and evaporation of the solvent, the residue was dissolved in 1 mL deuterated chloroform.

#### 3.4.2. Methanol Extraction

Ground lichens were extracted in 5 mL methanol for 20 min at 65 °C or room temperature to obtain polar metabolites. After filtration of the reaction mixture and evaporation of the solvent, the residue was dissolved in 1 mL deuterated methanol. Each sample was extracted independently two or three times.

### 3.5. NMR Spectroscopy

570 µL of the extracts were transferred to a standard 5 mm NMR tube. ^1^H-NMR spectra and two-dimensional COSY, NOESY, HSQC, and HMBC were acquired with a Bruker AVANCE I (500.13 MHz) at 300 K using standard parameters and suppression of water signal. TopSpin 2.1 (Bruker, Karlsruhe, Germany) was used for the acquisition and processing of the spectra. All spectra were manually phased and calibrated to 0 ppm for the trimethylsilane signal (hexane extracts) or trimethylsilyl propionate signal (methanol extracts), respectively. Furthermore, baseline correction was conducted. For the measurement of reference compounds, 10 mg were dissolved in 1 mL deuterated methanol and the solution was vortexed for 2 min. Then, the solution was centrifuged at 7000 U/min for 5 min and 570 µL of the supernatant was transferred into a NMR tube and measured as described above.

### 3.6. PCA

Principal component analysis was performed with the ^1^H-NMR spectra of methanolic lichen extracts using *AMIX* version 3.9.13 (Bruker, Karlsruhe, Germany). For this purpose, the spectra were divided into equally sized buckets with a width of 0.05 ppm within the region of 12.0 ppm to 0.0 ppm using the advanced bucketing command. Each bucket was then integrated and a data matrix was constructed with each row representing a sample (*i.e.*, spectrum) and each column representing a variable (*i.e.*, bucket). Rows were scaled to total intensity and the resulting values were used for PCA. The regions between 4.87–4.80, 3.37–3.12, 2.17–2.12, and 1.50–0.80 ppm were excluded due to the presence of residual water, methanol, acetone, and solvent impurity signals, respectively. Mean PCA scores were calculated and graphed. Influential buckets in the PCA scores were analyzed using the 2D loadings plots and the peaks within these buckets were identified by comparison with reference data.

### 3.7. GC-MS

Equivalents (200 µL) of the methanolic extracts were dried under N_2_ flow at room temperature after addition of 10 µL 5 mM D-norvaline. Then, the samples were derivatized with 20 mg·mL^−1^ methoxyamine hydrochloride (in pyridine) at 40 °C for 1.5 h under shaking. Next, after another drying step using N_2_ flow, the samples were treated with 100 µL *N*-methyl-*N*-(trimethylsilyl)-trifluoroacetamide (with 1% trimethylchlorosilane; MSTFA) at 55 °C for 30 min shaking. The MSTFA solution was subjected to GC-MS analysis without further preparation. The measurements were performed on a GC 2010 Gas Chromatograph and a GCMS-QP 2010 Plus mass spectrometer coupled to a QP-5000 mass selective detector (Shimadzu, Duisburg, Germany) working with electron impact (EI) ionisation at 70 eV and scanning from *m/z* 50 to *m/z* 800. A Silica capillary column Equity TM-5 (30 m × 0.25 mm × 0.25 µm film thickness) from Supelco Inc. (Bellefonte, PA, USA) was used. An aliquot of derivatives of the metabolites was injected in 1:10 split mode at 260 °C and a helium inlet pressure of 76.1 kPa. The interface temperature was 260 °C and the helium column flow was 1.19 mL/min. The column was developed at 70 °C for 5 min and then with a temperature gradient of 5 °C/min to a final temperature of 340 °C that was held for 2 min. The system was operating the GC-MS solution Ver. 2 software (Shimadzu, Duisburg, Germany) and the identification of the metabolites was carried out by using the NIST05 and NIST05s mass spectral reference library (Shimadzu, Duisburg, Germany).

## 4. Conclusions

Metabolite fingerprinting by NMR-spectroscopy showed that a set of Ecuadorian lichens under study contained only few hydrophobic metabolites regardless of their species or habitat. On the other hand, polar metabolites were extracted in high concentrations and metabolite fingerprinting indicated that the polar metabolome is not, or at least not greatly, affected by the growth location. In contrast, genera, and even species, were clearly differentiated on the basis of metabolite profiles. Additionally, distinct phenotypes could be distinguished by their chemical fingerprints, which corresponded nicely to phylogenetic groups obtained in the analysis of nrITS DNA data.

Lichen-specific metabolites can also be directly assigned by careful analysis of two-dimensional NMR experiments using the crude lichen extracts as exemplified for the alkaloid sticticin. The example shows that the multivariate method can be combined with targeted analyses aimed at a facile and rapid assignment of major lichen compounds. In all lichens, characteristic sugars or polyols, which are presumably transferred from the photobiont to the fungus or formed immediately from transferred carbon compounds, were present as shown by the non-targeted and targeted analyses.

The method combination described in this study appears generally valid for a chemo-taxonomic analysis of lichens. Moreover, the non-targeted approach could benefit the rapid discovery of hitherto unidentified lichen metabolites, at least of the major compounds present in crude extracts, on a systematic basis.
